# RNA-binding protein Musashi2 induced by RANKL is critical for osteoclast survival

**DOI:** 10.1038/cddis.2016.213

**Published:** 2016-07-21

**Authors:** T Fujiwara, J Zhou, S Ye, H Zhao

**Affiliations:** 1 Center for Osteoporosis and Metabolic Bone Diseases, Division of Endocrinology and Metabolism, Department of Internal Medicine, University of Arkansas for Medical Sciences, Little Rock, AR, USA

## Abstract

The Musashi family of RNA-binding proteins, Musashi1 and Musashi2, regulate self-renewal and differentiation of neuronal and hematopoietic stem cells by modulating protein translation. It has been recently reported that Musashi2, not Musashi1, regulates hematopoietic stem cells. Although osteoclasts are derived from hematopoietic cells, the expression and functions of Musashi proteins in osteoclast lineage cells remain unknown. In this study, we have uncovered that Musashi2 is the predominant isoform of Musashi proteins in osteoclast precursors and its expression is upregulated by receptor activator of NF-*κ*B ligand (RANKL) during osteoclast differentiation. Knocking down the expression of Musashi2 in osteoclast lineage cells by shRNAs attenuates nuclear factor of activated T cells 1 (NFATc1) expression and osteoclast formation *in vitro*. Mechanistically, loss of Musashi2 inhibits Notch signaling during osteoclast differentiation and induces apoptosis in pre-osteoclasts. In contrast, depletion of Musashi2 has no effects on cell cycle progression and p21^WAF-1^ protein expression in macrophages. Furthermore, depletion of Notch2 and its downstream target Hes1 in osteoclast precursors by shRNAs abrogates osteoclastogenesis by inhibiting NFATc1. Finally, absence of Musashi2 in osteoclast precursors promotes apoptosis and inhibits RANKL-induced nuclear factor-*κ*B (NF-*κ*B) activation, which is essential for osteoclast survival, Thus, Musashi2 is required for cell survival and optimal osteoclastogenesis by affecting Notch signaling and NF-*κ*B activation.

Skeletal homeostasis in adults is regulated by two opposing but equilibrating processes, bone resorption by osteoclasts and bone formation by osteoblasts. In the pathological settings, however, excessive bone resorption, either caused by increased osteoclast number or enhanced activity, leads to bone loss in metabolic bone diseases such as postmenopausal osteoporosis, rheumatoid arthritis, Paget's disease of bone, periodontal disease and lytic tumor bone metastasis.^1^

Osteoclasts are multinucleated cells formed by fusion of mononuclear precursors of the monocyte/macrophage lineage of hematopoietic origin. Macrophage colony-stimulating factor (M-CSF) and the receptor activator of nuclear factor-*κ*B (NF-*κ*B) ligand (RANKL) are two indispensable cytokines for osteoclastogenesis *in vitro* and *in vivo*. Deficiency of these two cytokines or their cognate receptors eliminates osteoclast formation and causes severe osteopetrosis in humans and rodents.^2, 3, 4, 5, 6^ Although M-CSF stimulates the proliferation of macrophages and the survival of osteoclasts by activating extracellular signal-regulated kinase (ERK) and phosphoinositide-3-kinase/Akt (PI3K/AKT) pathways, RANKL is a major, if not the only, osteoclast differentiation factor. RANKL activates mitogen-activated protein kinase (MAPK), NF-*κ*B, and PI3K/AKT pathways and induces calcium oscillation that also requires co-stimulating signals from immunoglobulin-like receptors and their associated adapter proteins.^7^ These pathways converge to induce and activate a transcriptome, consisting of nuclear factor of activated T cells 1 (NFATc1), activator protein-1, PU.1, microphthalmia-associated transcription factor and cAMP response element-binding protein, that regulates the expression of osteoclast-specific genes.^8, 9^ In addition to activating these positive regulators, RANKL has recently been shown to stimulate osteoclast differentiation by repressing the expression of negative osteoclastogenic genes such as interferon regulatory factor-8 (Irf8), B-cell lymphoma 6 (Bcl6) and v-Maf musculoaponeurotic fibrosarcoma oncogene family member protein B (MafB), through the induction of B lymphocyte-induced maturation protein-1 (Blimp1) and DNA methyltransferase 3a (Dnmt3a).^10, 11, 12, 13, 14^

Notch signaling is an evolutionarily conserved pathway that is critical for cell fate determination during development and adult tissue homeostasis by regulating cell proliferation, differentiation and apoptosis.^15^ In mammals, there are four Notch receptors (Notch1–4) and at least five classical ligands (Delta-like-1, -3, -4 and Jagged-1 and -2). Both Notch receptors and ligands are single-pass transmembrane proteins localized at the cell surface. Interaction of Notch receptor with a ligand on adjacent cell triggers two sequential proteolytic cleavages mediated by a disintegrin and metalloproteinase domain-containing protein 10 (ADAM 10) and a *γ*-secretase complex, respectively. As a consequence, the Notch intracellular domain (NICD) is released from the plasma membrane and translocates to the nucleus where it forms a ternary complex with DNA-binding protein, recombinant recognition sequence binding protein at the J*κ* site (RBPJ*κ*), and co-activator, a mastermind-like protein, to induce the transcription of Notch target genes, including members of hairy and enhancer of split (Hes) and Hes related with YRPW motif (Hey) families.^16^ In addition to this RBPJ*κ*-dependent, canonical Notch signaling, NICD has been reported to interact with components of other signaling pathways, such as NF-*κ*B and Wnt, thus exerts RBPJ*κ*-independent, non-canonical effects on a variety of cellular processes.^17^

Inherited loss and gain of function of Notch receptors and their ligands in humans have been linked to skeletal disorders such as spondylocostal dysostoses, Alagille syndrome, Hajdu-Cheney syndrome and osteosarcoma.^18, 19, 20, 21^ The recent mouse genetic studies have elucidated that Notch signaling promotes the proliferation of mesenchymal progenitors and early osteoblasts but inhibits their terminal differentiation to mature osteoblasts to help maintain bone marrow mesenchymal stem cells.^22, 23, 24^ Therefore, loss of Notch signaling in osteoblast lineage cells increases bone mass in young mice and causes osteopenia in aged mice due to exhaustion of mesenchymal stem cells and increased osteoclasts. In contrast, the intrinsic role of Notch signaling in osteoclast lineage cells remains obscure. Both inhibitory and stimulatory effects of Notch signaling on osteoclastogenesis have been reported. Although Notch1- and RBPJ*κ*-dependent canonical pathway inhibit osteoclast differentiation,^25, 26, 27^ Notch2 promotes RANKL-induced osteoclastogenesis by its association and activation of NF-*κ*B.^28^ Notch activation at different stages of osteoclast differentiation (uncommitted macrophages versus RANKL-primed osteoclast precursors), by different ligands (Delta-like-1 versus Jagged-1), or by different receptors (Notch1 versus Notch2) exerts distinct effects on osteoclast formation *in vitro*.^29, 30^ Thus, it seems that Notch signaling regulates osteoclastogenesis in receptor/ligand-, cell stage- and downstream pathway-specific manners. Hence, characterization of the cell-autonomous functions of Notch signaling in osteoclast lineage cells demands further precise investigations using both genetically modified animal models and *in vitro* cell culture system.

Among Notch modulators, mammalian Numb and Numb-like have been shown to inhibit Notch signaling by regulating NICD endosomal trafficking, degradation and nuclear translocation.^31, 32, 33^ More recently, Numb and Numb-like have been reported to regulate Notch signaling and RANKL-induced osteoclastogenesis in mice.^34^ The Musashi (Msi) family of evolutionary conserved RNA-binding proteins, including Msi1 and Msi2 in mammals, repress translation initiation by binding consensus motifs in the 3′ untranslated regions of the target mRNAs.^35^ Although Msi1 is restricted to neuronal stem cells and regulates their self-renewal, Msi2 is predominantly expressed in hematopoietic stem cells (HSC) and regulates normal hematopoiesis.^36, 37^ Two major targets of Msi identified so far are cell cycle inhibitor p21^WAF-1^ and Numb.^38, 39, 40, 41^ Aberrant overexpression of Msi2 predisposes to aggressive myeloid leukemia. Msi2 and Notch signaling are upregulated in the course of human myeloid leukemia progression.^42, 43, 44, 45^

Osteoclasts are derived from myeloid lineage of HSC, but the expression and function of Msi, especially Msi2, in osteoclast lineage cells remain undefined. Here we report that Msi2 is the predominant isoform of Msi in osteoclast precursors and its expression is upregulated by RANKL. Loss of Msi2 in osteoclast precursor cells attenuates the expression of Notch2/Hes1 and RANKL-induced NF-*κ*B activation, leading to promote apoptosis and inhibit osteoclast differentiation *in vitro*. Thus, Msi2 induced by RANKL is a novel regulator of osteoclast survival via affecting Notch signaling and NF-*κ*B activation.

## Results

### Msi2 is highly expressed in osteoclasts and its deletion attenuates osteoclastogenesis *in vitro*

**Figure 1:**
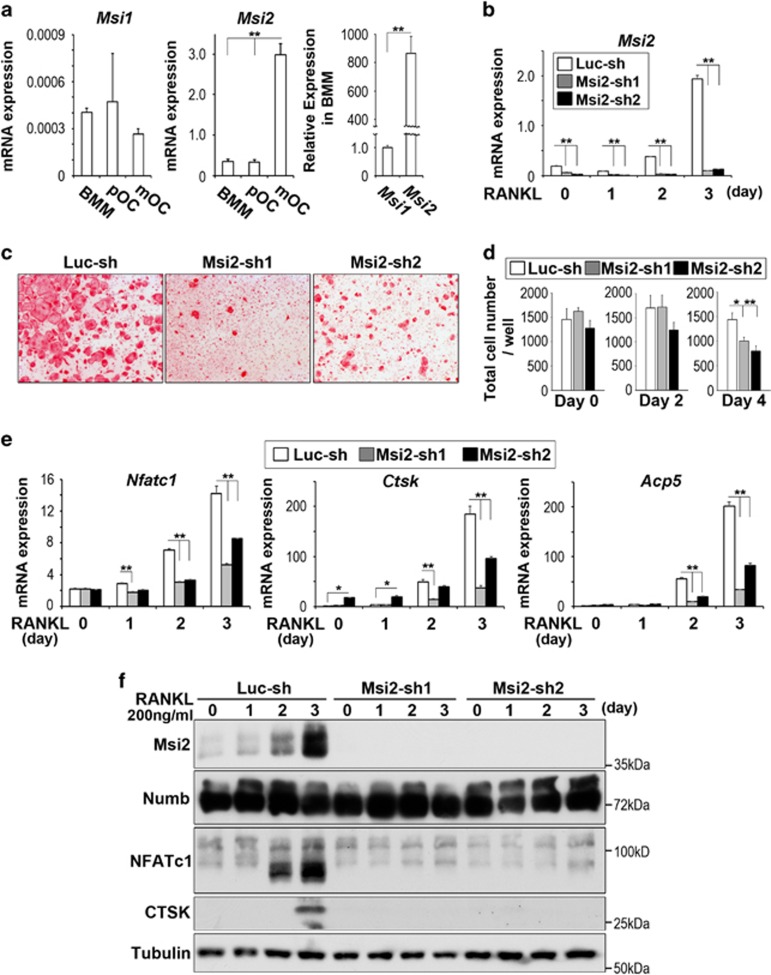
Msi2 is critical for osteoclastogenesis *in vitro*. (**a**) BMM were cultured with M-CSF, or with both M-CSF and RANKL for 2 and 4 days to generate pre-osteoclasts (pOC) and mature osteoclasts (mOC), respectively. The levels of mRNA expression of *Msi1* (left) and *Msi2* (center) were measured by real-time PCR. (**b**) After knocking down BMM by *Msi2*-shRNAs (Msi2-sh1 or -sh2) or a control shRNA (Luc-sh), each cells were induced osteoclasts for 3 days. The levels of *Msi2* expression were quantified by real-time PCR. (**c**) TRAP staining was performed after culture with M-CSF and RANKL for 4 days. (**d**) After each cells (Luc-sh and Msi2-sh1 and sh2) were cultured with M-CSF, or with both M-CSF and RANKL for 2 and 4 days at 96-well plate, total cell numbers in each stage (day 0, 2 and 4) were counted. (**e**) The levels of mRNA expression of osteoclast markers, NFATc1 (*Nfatc1*); Cathepsin K (*Ctsk*); and TRAP (*Acp5*) were measured by real-time PCR. (**f**) Protein expressions of osteoclast markers were detected by western blotting. Tubulin served as a loading control. A two-tailed Student's *t*-test was used for 2-group comparisons. Multiple samples were analyzed by one-way ANOVA (*P<0.05; **P<0.01)

In an attempt to elucidate the role of Msi RNA-binding proteins in osteoclast lineage cells, we first measured mRNA levels of *Msi1* and *Msi2* during the course of osteoclast differentiation by real-time quantitative PCR. For this purpose, bone marrow macrophages (BMMs) were cultured with M-CSF alone (BMM) or with a combination of M-CSF and RANKL for 2 and 4 days to generate pre-osteoclasts (pOC) and mature osteoclasts (mOC), respectively. As shown in Figure 1a, the mRNA level of *Msi2* in BMMs is over 800-fold higher than that of *Msi1*. Furthermore, both Msi2 mRNA and protein were expressed at low level in BMM but were markedly upregulated by RANKL during osteoclast differentiation (Figures 1a and c). These data indicate that Msi2 is the predominant isoform of Msi RNA-binding proteins in osteoclasts and their precursors. We next set out to determine the cell-autonomous functions of Msi2 in osteoclast differentiation and/or function. To this end, we knocked down Msi2 expression in BMMs by lentiviral transduction of two short hairpin (sh) RNAs (Msi2-sh1 and Msi2-sh2), targeting different sites of murine *Msi2* mRNA. A shRNA targeting firefly luciferase (Luc-sh) was used as a negative control. Positively transduced BMMs were either cultured with M-CSF alone or stimulated with M-CSF and RANKL for 3 days. Both Msi2-specific shRNAs, but not Luc-sh, markedly reduced *Msi2* mRNA (Figure 1b) and protein (Figure 1f) expression in BMMs and RANKL-induced osteoclast precursors, as detected by real-time PCR and western blots, respectively. Depletion of Msi2 in osteoclast precursor cells attenuated osteoclast formation as demonstrated by decreased number of multinucleated osteoclasts staining positively by tartrate-resistant acid phosphatase (TRAP), an osteoclast differentiation marker (Figure 1c). The total cell number at different stages of control and Msi2-depletion cultures were counted and found significantly decreased only at day 4 (Figure 1d). The abrogated osteoclastogenesis was further confirmed by decreased mRNA expression of osteoclast marker genes, such as *NFATc1* (encoded by *Nfatc1*), *Cathepsin K* (encoded by *Ctsk*) and *TRAP* (encoded by *Acp5*) in Msi2-deficient osteoclast precursors as compared with control cells, measured by real-time PCR (Figure 1e). Moreover, RANKL-induced protein expression of NFATc1 and CTSK was greatly reduced in Msi2-depleted cells (Figure 1f). Numb is one of the targets of Msi proteins reported so far and Numb/Numb-like regulate RANKL signaling and osteoclastogenesis.^34, 46^ We thus examined Numb protein expression in RANKL-stimulated control and Msi2-depleted osteoclast precursor cells. Both 72 and 74 kd isoforms of Numb were detected by western blotting with the former being abundant in osteoclast precursors. Increased Msi2 during osteoclastogenesis did not affect Numb expression, and loss of Msi2 had no effect on the levels of Numb compared to control cells (Figure 1f). Taken together, Msi2 is induced by RANKL and positively regulates osteoclastogenesis *in vitro*, probably through Numb-independent mechanisms.

### Msi2 regulates Notch signaling and is critical for osteoclast survival

**Figure 2:**
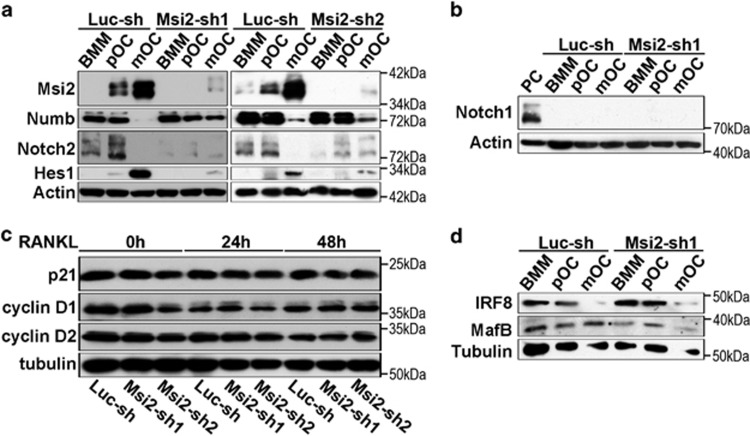
Knocking down Msi2 attenuated Notch2/Hes1, not cell cycle regulators. (**a**) The protein of Msi2, Numb, NICD2 and Hes1 in control (Luc-sh) and Msi2 knocking down (Msi2-sh1 and sh2) cells was detected by western blots. Actin served as a loading control. (**b**) Notch1 was undetectable in both cells. A human Merkel cell carcinoma cell was used as a positive control (PC). (**c** and **d**) Western blots of cell cycle regulators, p21^WAF1^ and cyclin D1/D2, and the negative regulators of osteoclastogenesis, Irf8 and MafB. Tubulin served as loading controls

Msi proteins have been reported to activate Notch signaling through the translational repression of Numb, an evolutionarily conserved NICD antagonist^39, 41^ and to regulate cell cycle progression by translational inhibition of the cyclin-dependent kinase inhibitor p21^WAF1^. ^38^^–^^40^ As both Notch and p21/cyclin D pathways are critical for osteoclast formation,^34, 46, 47^ we therefore asked whether inhibition of Msi2 could have impact on these two pathways in osteoclast lineage cells. To answer this question, we first examined the protein levels of the components of these two pathways in control and Msi2-deficient BMMs and osteoclasts. As shown in Figure 2a, the protein expression of Msi2 was greatly inhibited by two shRNAs during osteoclast differentiation. In consistence with the result shown in Figure 1f, loss of Msi2 led to a slight increase of Numb accumulation in mature osteoclasts but had no effect on Numb translation and protein levels in BMMs and pre-osteoclasts. Together with the recent reports in other cell types,^37, 48, 49^ these data (Figures 1f and 2a) indicate that Numb may not be a direct target of Msi2 in osteoclast precursors. On the other hand, depletion of Msi2 diminished NICD2 and its downstream target Hes1 in BMMs and osteoclasts. In accordance with our previous reports,^46, 50^ Notch2 was highly activated in osteoclast precursors and its activation was markedly decreased in mature osteoclasts, suggesting that Msi2 may regulate Notch2 expression in osteoclast precursor cells. Besides, both Msi2 and Hes1 were highly expressed in mature osteoclasts and loss of Msi2 abolished Hes1 expression in these cells. These data suggest that Msi2 may directly regulate Hes1 as reported by Szabat *et al.*^48^ As reported in our previous work,^50^ Notch1 was undetectable in BMMs and osteoclasts (Figure 2b). In contrast to leukemic cells, depletion of Msi2 in BMMs had little effects on p21^WAF1^ and cyclin D1/D2 (Figure 2c). Accumulating evidence indicates that transcriptional repression of negative osteoclastogenic genes such as Irf8, MafB and Bcl6 by RANKL-induced Blimp1 and Dnmt3a represents a key mechanism regulating osteoclast differentiation.^9^ Therefore, Msi2 may suppress the expression of these osteoclastogenic inhibitors. To test if such is a case, we detected the protein levels of Irf8 and MafB, in control and Msi2-depleted BMMs and osteoclasts by western blots, because no reliable anti-Bcl6 antibodies were available at the moment. Figure 2d showed that the expression pattern of these proteins was indistinguishable in control and Msi2 knockdown cells.

**Figure 3:**
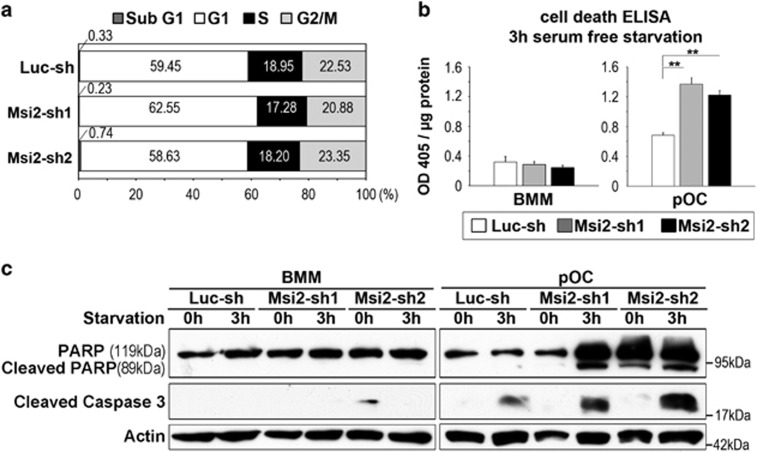
Loss of Msi2-induced apoptosis in pre-osteoclasts. (**a**) Cell cycle progression in BMM was measured by flow cytometry using 7′AAD. The average percentage of cell number in each phase of cell cycle is shown in the graphs. (**b**) The control and Msi2 knockdown BMM or pOC were serum/cytokine starved for 3 h. The apoptosis rate was measured by a cell death ELISA. Difference was analyzed by one-way ANOVA (*versus* Luc-sh) (**P<0.01) (**c**) Luc-sh and Msi2-sh1 and sh2 BMM or pOC were either untreated or serum/cytokine starved for 3 h. The cleaved caspase 3 and PARP, two markers of apoptosis, was detected by western blots. Actin served as a loading control

Next, we tried to identify which cellular processes affected by Msi2 downregulation in osteoclast lineage cells. Consistent with the result presented in Figure 2c, loss of Msi2 did not affect cell cycle progression in BMMs (Figure 3a), as measured by flow cytometry. In addition, deletion of Msi2 in BMMs had little effect on apoptosis induced by cytokine/serum-starvation, determined by a cell death ELISA and western blotting of cleaved (active) Caspase 3 and poly (ADP-ribose) polymerase (PARP), two mediators and markers of apoptosis (Figures 3b and c, left panels). In contrast, depletion of Msi2 in pre-osteoclasts accelerated apoptosis under the same condition (Figures 3b and c, right panels). Taken together, these results indicate that Msi2 regulates Notch signaling and Hes1 in osteoclasts and enhances osteoclastogenesis by promoting survival of osteoclasts.

### Notch2 and Hes1 are critical for osteoclastogenesis *in vitro*

**Figure 4:**
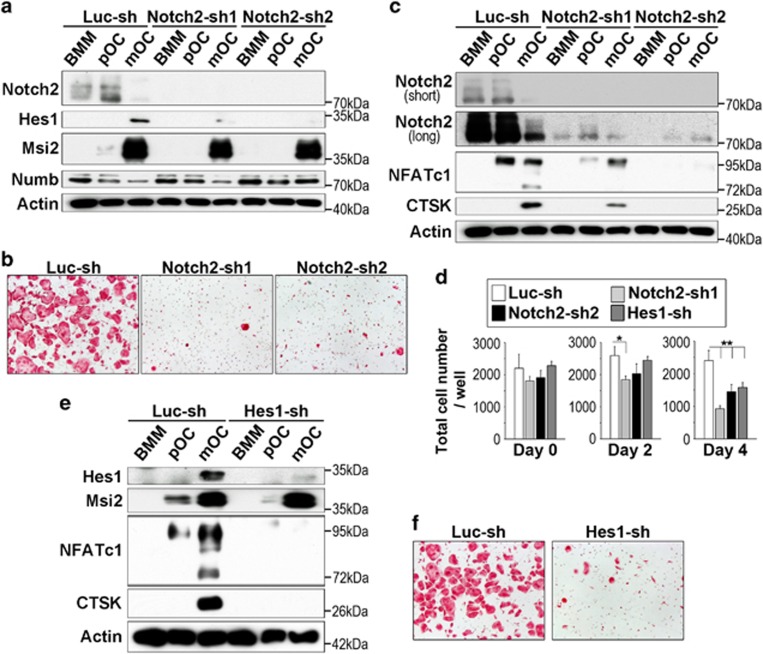
Knocking down the expression of Notch2 and Hes1 attenuated osteoclast formation. (**a**) The protein levels of NICD2, Hes1, Msi2 and Numb in control and Notch2 knockdown osteoclast lineage cells was detected by western blots. Actin served as a loading control. (**b**) TRAP staining of control and Notch2 knockdown cultures. (**c**) The protein expression of NICD2 (upper: short exposure, lower: long exposure) and osteoclastic markers, NFATc1 and CTSK, was detected by western blots. Actin served as a loading control. (**d**) After each cells (Luc-sh, Notch2-sh1 and sh2 and Hes1-sh) were cultured with M-CSF, or with both M-CSF and RANKL for 2 and 4 days at 96-well plate, total cell numbers in each stage (day 0, 2 and 4) were counted. Difference was analyzed by one-way ANOVA (*versus* Luc-sh) (*P<0.05, **P<0.01). (**e**) Western blots of Hes1, Msi2, NFATc1, and CTSK expression in control and Hes1 knocking down cultures. Actin served as a loading control. (**f**) TRAP staining of control and Hes1 knockdown osteoclast cultures

The results presented so far suggest that Msi2 promotes osteoclast formation by upregulating Notch signaling and Hes1 in osteoclast precursors. Given that both inhibitory and stimulatory effects of Notch activation on osteoclastogenesis have been reported, we next tested whether knocking down Notch2 in BMMs would result in decreased osteoclast formation similar to that of Msi2 depletion. As demonstrated in Figure 4a, the expression of Notch2 and its downstream target Hes1 was greatly reduced by two Notch2-specific shRNAs. Notch2-sh2 seemed more potent than Notch2-sh1 (see longer exposure of Notch2 immunoblot; Figure 4c). Because Msi2 acts upstream of Notch signaling, its expression was not affected by decreased Notch2 expression. Loss of Notch2 in osteoclast lineage cells led to slightly increased Numb level, probably caused by loss of a negative feedback action of Notch signaling on Numb and Numb-like.^41^ Similar to Msi2 depletion, decreased Notch2 expression in BMMs abrogated osteoclast differentiation, as demonstrated by less number of TRAP-positive multinucleated osteoclasts and decreased protein levels of NFATc1 and CTSK in Notch2-depleting cultures compared with the control (Figures 4b and c). Again, Notch2-sh2 showed more potent inhibition than Notch2-sh1 in terms of NFATc1 and CTSk expression (Figure 4c). Likewise, knocking down the expression of Hes1 in osteoclast lineage cells blocked osteoclast differentiation without changing the level of Msi2 (Figures 4e and f). As is the case with Msi2 knockdown cells, total cell number of each knockdown cells (Notch2-sh1 and 2, and Hes1-sh) were reduced at late stages of osteoclast cultures (Figure 4d). These data indicate, but do not directly prove, that Msi2 regulates osteoclastogenesis, in part, through Notch2 and Hes1.

### Msi2 modulates RANKL-induced NF-*κ*B activation in pre-osteoclasts

**Figure 5:**
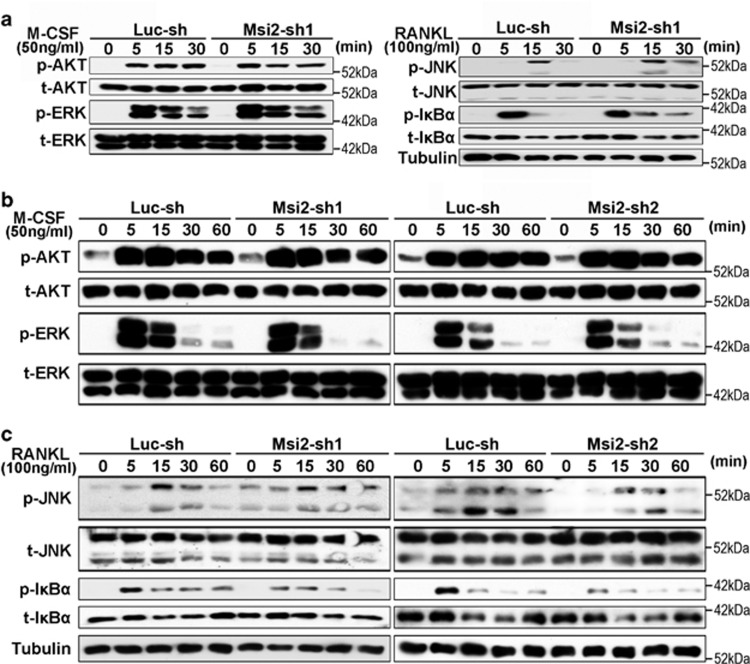
Msi2 is required for RANKL-induced NF-*κ*B activation in pre-osteoclasts. Western blotting detection of the signaling pathways downstream of M-CSF or RANKL in BMMs (**a**) and pOC (**b** and **c**). Tubulin served as loading controls

Signaling pathways activated by M-CSF and RANKL are indispensable for the proliferation, survival and differentiation of osteoclast precursor cells.^7^ Lastly, we determined whether loss of Msi2 in BMMs and pre-osteoclasts could affect M-CSF and/or RANKL signaling pathways. For this purpose, control and Msi2 knockdown BMMs and pre-osteoclasts were serum and cytokine starved and then stimulated with M-CSF or RANKL for the indicated time. The activation of M-CSF-induced ERK and AKT (Ak strain transforming, also known as protein kinase B), and RANKL-induced c-Jun N-terminal kinase (JNK) and NF-*κ*B was detected by western blots. As demonstrated in Figure 5a, decreased expression of Msi2 in BMMs had no effects on the activation of both M-CSF and RANKL signaling pathways. Deletion of Msi2 in pre-osteoclasts by both Msi2-shRNAs exerted little effects on ERK and AKT activation upon M-CSF stimulation (Figure 5b). Although knocking down Msi2 in pre-osteoclasts by Msi2-sh1 caused a marginal increase in prolonged JNK activation in response to RANKL (Figure 5c), however, this effect was not observed in Msi2-sh2-expressing cells, indicating that Msi2 may not directly regulate JNK activation in osteoclast lineage cells. In contrast, downregulation of Msi2 by both shRNAs consistently decreased RANKL-induced NF-*κ*B activation as demonstrated by reduced level of phospho-I*κ*B (Figure 5c). Given that NF-*κ*B pathway plays an essential role in osteoclast survival,^51^ the cellular process regulated also by Msi2 (Figures 3b and c), these results indicate that Msi2 promotes osteoclast survival through modulating RANKL-induced NF-*κ*B activation.

## Discussion

**Figure 6:**
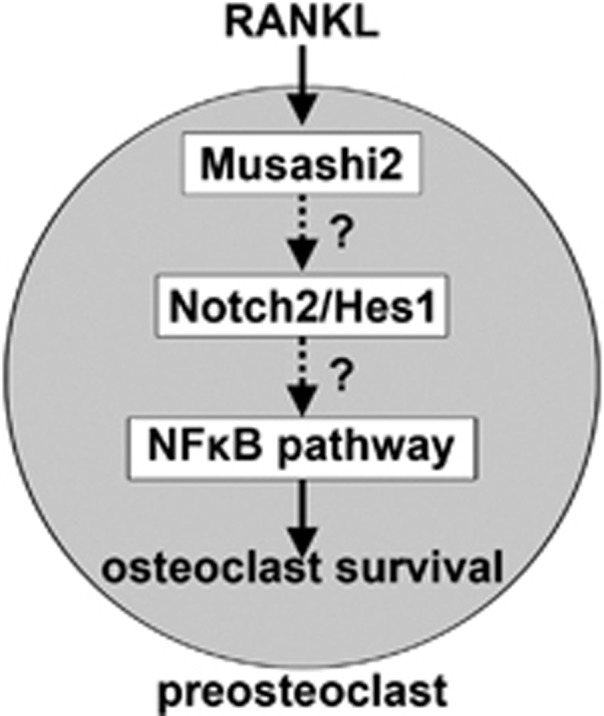
A schematic model of Msi2 in osteoclastogenesis

The Msi family of RNA-binding proteins are evolutionary conserved cell fate determinants that have an essential role in development and tissue homeostasis by regulating self-renewal and terminal differentiation of neuronal and other tissue stem cells.^52^ Of particular relevant to osteoclast biology, Msi2 is predominantly expressed in HSC and regulates normal hematopoiesis. The aberrant activation of Msi2 is associated with aggressive myeloid leukemia.^43, 44^ As osteoclasts are derived from the myeloid lineage of HSC, it is likely that Msi2 is also critical for osteoclast formation and/or function. However, the role of Msi2 in osteoclast lineage cells has not been elucidated so far. In the present study, we have uncovered that Msi2, but not Msi1, is highly induced by RANKL during osteoclast differentiation. Msi2 regulates Notch2 activation and Hes1 in osteoclast precursors and is critical for osteoclast differentiation and survival by modulating RANKL-induced NF-*κ*B activation (Figure 6).

Msi proteins were originally identified as regulators of cell cycle progression and differentiation by their translational repression of cyclin-dependent kinase inhibitor p21^WAF1^ and Notch antagonist Numb.^35^ As both Numb/Notch and p21/cyclin pathways are involved in osteoclastogenesis, we postulated that Msi2 regulates osteoclast formation by targeting mRNAs of p21^WAF1^ and Numb in osteoclast lineage cells. Depletion of Msi2 in osteoclast precursor cells, however, had no effects on the levels of p21/cyclin D1/2 and did not affect cell cycle progression in macrophages (Figures 2c and 3a). Although Numb protein expression was shown to decrease in control mature osteoclasts which had abundant Msi2, depletion of Msi2 in BMMs and pre-osteoclasts had little effect on Numb mRNA translation and protein level (Figures 1f and 2a). Therefore, p21^WAF1^ and Numb may not be the direct RNA targets of Msi2 in osteoclast lineage cells. In support of this notion, a recent high-throuput sequencing and cross-linking immunoprecipitation (HITS-CLIP) study in K562 leukemia cells have revealed that the direct RNA-binding targets of Msi2 are associated with a variety of pathways involving in RNA metabolism/translation and TGF-*β* signaling, independently from Numb.^37^ The targets of Msi2 in osteoclast lineage cells need to be further identified in the future.

Loss of Msi2 eliminated Notch2 activation in BMMs and pre-osteoclasts and reduced Hes1 expression in pre-osteoclasts and mature osteoclasts (Figure 2a). Moreover, downregulation of all three proteins by specific shRNAs in osteoclast lineage cells abrogated osteoclast differentiation (Figures 1 and 4). These data indicate, but not directly prove, that Msi2 may regulate osteoclastogenesis through Notch2 and/or Hes1 in osteoclast precursors. The rescue of osteoclast formation defect by overexpression of Notch2 or Hes1 in Msi2 knocking down cells will provide direct evidence to prove this hypothesis. However, there is a technical challenge at the moment for conducting such experiment because primary BMMs could not survive by two rounds of viral transduction (unpublished data). Future experiments using Msi2-null macrophages isolated from Msi2 knockout mice will be very helpful. More recently, it has been reported that Notch2 is one of the high-confidence Msi2 targets in epithelial cells identified by HITS-CLIP.^53^ It will be necessary to determine whether Msi2 binds to Notch2 RNA and regulates its expression in osteoclast lineage cells using the similar techniques in the future.

Downregulation of Msi2 in osteoclast lineage cells by specific shRNAs diminished NFATc1 induction during osteoclast differentiation (Figure 1f), suggesting that Msi2 is required for the induction and activation of this critical osteoclastogenic transcription factor. In addition to co-activating signals derived from immunoglobulin-like receptors and their associated adapter proteins, RANKL induces and activates NFATc1 by repressing the expression of negative osteoclastogenic genes such as Irf8 and MafB. As a translational regulator, Msi2 may decrease the protein levels of these inhibitory factors of osteoclastogenesis. However, depletion of Msi2 in osteoclast lineage cells did not changes the protein expression of Irf8 and MafB. In contrast, inhibition of Notch2 or Hes1 expression by shRNAs markedly reduced NFATc1 induction (Figures 4c and e) during osteoclast differentiation, indicating that Msi2 may regulate NFATc1 through Notch2/Hes1/NF-*κ*B pathway. It has been recently reported that Notch/RBPJ*κ*/Hey1 axis directly inhibits NFATc1 in osteoblasts.^54^ As described above, Notch signaling, especially Notch2, seems to positively regulate NFATc1. Thus, Notch signaling regulates NFATc1 in cell-type-specific and downstream pathway-specific manners. How Notch signaling induces NFATc1 in osteoclast lineage cells needs further investigation.

It should be pointed out that the cell-autonomous functions of Notch signaling in osteoclasts remain unclear. Although Notch1 and RBPJ*κ*-dependent canonical pathway have been reported to suppress osteoclast differentiation,^25, 26, 27^ we and others have found that Notch2 is required for osteoclastogenesis and survival.^28, 30, 46, 50^ The mechanistic explanations for this discrepancy are unknown. Previous work by others has shown that activation of Notch2/Hes1 is accompanied by increased NF-*κ*B activity in osteoclast and B chronic lymphocytic leukemia.^28, 55^ It has also been reported that Notch signaling activates NF-*κ*B pathway in several cell types.^56, 57, 58, 59, 60^ Moreover, Hes1, a well-identified target of Notch signaling,^16, 61^ has been demonstrated to induce NF-*κ*B activation directly in 293 T cells.^62^ Given the critical role of NF-*κ*B pathway in osteoclast survival and differentiation^51^ and the suppressive effects of *γ*-secretase inhibitors on osteoclastogenesis,^29, 30^ the data presented in this study indicate that Notch2/Hes1 pathway positively regulates osteoclast differentiation and survival by affecting NF-*κ*B activity.

On the basis of the findings by this study and those reported in the literature, we propose that Msi2 is induced by RANKL during osteoclast differentiation and that Msi2 activates Notch2 and Hes1, which lead to the activation of NF-*κ*B and NFATc1. Hence, Msi2 is required for optimal osteoclast differentiation and survival (Figure 6).

## Materials and Methods

### Antibodies and reagents

Antibodies were obtained from the following resources: mouse monoclonal anti-beta actin (GeneScript, Piscataway, NJ, USA, A00702); rabbit monoclonal anti-cleaved caspase 3 (Cell Signaling Technology, Beverly, MA, USA, 9664); mouse monoclonal anti-Cathepsin K (Millipore, Temucula, CA, USA, clone 182-12G5); rabbit polyclonal anti-cyclin D1 (Cell Signaling Technology, 2922); rabbit polyclonal anti-cyclin D2 (Santa Cruz Biotechnologies, Dallas, TX, USA, sc-181); rabbit polyclonal anti-Hes1 (Abcam, Cambridge, MA, USA, ab157181); rabbit monoclonal anti-Irf8 (Cell Signaling Technology, 5628); rabbit polyclonal anti-MafB (Abcam, ab65953) and rabbit monoclonal anti-Msi2 (Abcam, ab76148); mouse monoclonal anti-NFATc1 (Santa Cruz Biotechnology, sc-7294); rat monoclonal anti-Notch1 (bTAN 20) and anti-Notch2 (C651.6DbHN) (Developmental Studies Hybridoma Bank, Iowa City, Iowa, USA); rabbit monoclonal anti-Numb (2756) and rabbit polyclonal anti-PARP (9542) (Cell Signaling Technology); mouse monoclonal anti-*α*-tubulin (Sigma-Aldrich, St. Louis, MO, USA, clone DM1A); rabbit monoclonal anti-phosphor-Akt (4058), mouse monoclonal anti-Akt (2920), mouse monoclonal anti-phospho-ERK1/2 (9106), rabbit polyclonal anti-ERK1/2 (9102), mouse monoclonal anti-phospho-IКB-*α* (9246), rabbit polyclonal anti-IКB-*α* (9242), mouse monoclonal anti-phospho-JNK (9255), rabbit polyclonal anti-JNK (9252), anti-phospho-p38 (9215), anti-total p38 (9212). *α*-minimal essential medium (*α*-MEM) (Life Technologies, Carlsbad, CA, USA), 10 × Penicillin–Streptomycin–l-Glutamine (Sigma-Aldrich), and fetal bovine serum (Hyclone, Carlsbad, CA, USA).

### Animal use approval

All animal procedures were approved by Institutional Animal Care and Use Committee at University of Arkansas for Medical Sciences. The methods were carried out in accordance with the approved guidelines.

### Bone marrow macrophage and osteoclast cultures

BMMs were prepared as described previously.^46^ Briefly, whole bone marrow was extracted from tibia and femurs of one or two 8–10-week-old C57/BL6J mice. Red blood cells were lysed in buffer (150 mM NH_4_Cl, 10 mM KNCO_3_, 0.1 mM EDTA, pH 7.4) for 5 min at room temperature. Bone marrow cells (5 × 10^6^) were plated onto a 100 mm petri-dish and cultured in *α*-10 medium (*α*-MEM, 10% heat-inactivated fetal bovine serum, 1 × Penicillin–Streptomycin–l–Glutamine solution) containing 1/10 volume of CMG 14–12 (conditioned medium supernatant containing recombinant M-CSF at 1 *μ*g/ml^63^) for 4 to 5 days. Fresh media and CMG 14-12 supernatant were replaced every the other day. Pre-osteoclasts and osteoclasts were generated after three and five days culture of BMMs (at density of 160/mm) with 1/100 vol of CMG 14–12 culture supernatant and 100 ng/ml of recombinant RANKL, respectively.

### Lentivirus mediated shRNA expression

The LKO.1 lentiviral vectors expressing shRNA sequence targeting mRNA of murine *Msi2* [TRCN0000071973/NM_054043.2-1537s1c1 (Msi2-sh1) and TRCN0000071974/NM_054043.2-797s1c1 (Msi2-sh2)], murine *Notch2* [TRCN00000340512/NM_010928.2-923s21c1 (Notch-sh1) and TRCN00000340513/NM_010928.2-4881s21c1 (Notch2-sh2)] were purchased from Sigma-Aldrich. A murine *Hes1* mRNA targeting shRNA (Hes1-sh) pair: CCGGGGCAGACATTCTGGAAATGACTCGAGTCATTTCCAGAATGTCTGCCTTTTTG/AATTCAAAAAGGCAGACATTCTGGAAATGACTCGAGTCATTTCCAGAATGTCTGCC was synthesized (Integrated DNA Technologies) and ligated into the AgeI/EcoRI site of the LKO.1 vector. A firefly luciferase shRNA was used as a control (5′-GCTTACGCTGAGTACTTCGA-3′). 293-T cells were co-transfected with a LKO.1 gene transfer vector and virus packaging vectors, ΔH8.2 and VSVG by TransIT-LT1 transfection reagent (Mirus). Virus supernatants were collected after 48 h transfection. BMMs were transduced with virus supernatant containing M-CSF and 20 *μ*g/ml of Protamine (Sigma-Aldrich). Cells were then selected in *α*-10 medium containing M-CSF and 6 *μ*g/ml puromycin (Sigma-Aldrich) for 3 days.

### TRAP staining

BMMs were cultured on 48-well tissue culture plate in *α*-10 medium with M-CSF and RANKL for 4-5 days. The cells were fixed with 4% paraformaldehyde/phosphate-buffered saline (PBS) and TRAP was stained with NaK Tartrate and Napthol AS-BI phosphoric acid (Sigma-Aldrich) as described previously.^50^

### RNA isolation and quantitative real-time RT-PCR

Total RNA was purified using RNeasy mini kit (Qiagen, Hilden, Germany) according to the manufacture's protocol. First-strand cDNAs were synthesized from 0.5–1 *μ*g of total RNA using the High Capacity cDNA Reverse Transcription kits (Life Technologies) following the manufacturer's instructions. TaqMan quantitative real-time PCR was performed using the following Primers from Life Technologies: *Acp5* (Mm00475698_m1); *Ctsk* (Mm00484039_m1); *Msi1* (Mm01203522_m1); *Msi2* (Mm01304232_m1); *Mrps2* (Mm03991065_g1); *Nfatc1* (Mm00479445_m1). Samples were amplified using the StepOnePlus real-time PCR system (Life Technologies) with an initial denaturation at 95 °C for 10 min, followed by 40 cycles of 95 °C for 15 s and 60 °C for 1 min. The relative cDNA amount was calculated by normalizing to that of the mitochondrial gene Mrps2, which is steadily expressed in both BMMs and osteoclasts, using the ΔCt method.^64^ The relative levels of *Msi1* and *Msi2* cDNAs in BMM were analyzed using the delta ΔCt method.

### Immunoblotting

Cultured cells were washed with ice-cold PBS twice and lysed in 1 × RIPA buffer (Sigma-Aldrich) containing 1 mM DTT and Complete Mini EDTA-free protease inhibitor cocktail (Roche, Indianapolis, IN, USA). After incubation on ice for 30 min, the cell lysates were clarified by centrifugation at 14 000 rpm for 15 min at 4 °C. Ten to thirty microgram of total protein were subjected to 8% SDS-PAGE gels and transferred electrophoretically onto polyvinylidene difluoride membrane (EMD Millipore) by a semi-dry blotting system (Bio-Rad, Hercules, CA, USA). The membrane was blocked in 5% fat-free milk/Tris-buffered saline for 1 h and incubated with primary antibodies at 4 °C overnight followed by secondary antibodies conjugated with horseradish peroxidase (Santa Cruz Biotechnology). After rinsing three times with Tris-buffered saline containing 0.1% Tween 20, the membrane was subjected to western blot analysis with enhanced chemiluminescent detection reagents (EMD Millipore, Billerica, MA, USA).

### Flow cytometric analysis of cell cycle progression

BMMs were lifted by 1 × Trypsin/EDTA (Life Technologies), resuspended in cold PBS at 1 × 10^6^/ml, and were fixed with 70% ethanol. Cells were incubated with 25 *μ*g/ml of 7-aminoactinomycin-D (7′AAD) (BD Biosciences) for 30 min on ice. The cell cycle analysis was performed by flow Cytometry.

### Cell death ELISA

For the detection of osteoclast apoptosis, BMMs were cultured with only M-CSF or with M-CSF and RANKL for 2 days. BMM and pre-osteoclasts were serum and cytokine starved for 3 h. Cell death was analyzed using cell death detection ELISA PLUS kit (Roche), which detects cytoplasmic histone-associated DNA fragmentation.

### Statistics

For all graphs, data are represented as the mean±S.D. Data of 2-group comparisons were analyzed using a two-tailed Student's *t*-test. For comparison of more than 2 groups, data were analyzed using one-way analysis of variance (ANOVA) and the Bonferroni procedure was used for Tukey comparison. Data in all graphs are represented as mean±S.D. *P*<0.05 was considered statistically significant.

## Abbreviations

AKT: Ak strain transforming, also known as protein kinase B
Bcl6: B-cell lymphoma 6
Blimp1: B lymphocyte-induced maturation protein-1
BMM: bone marrow macrophage
Dnmt3a: DNA methyltransferase 3a
ERK: extracellular signal-regulated kinase
Hes: hairy and enhancer of split
Hey: Hes related with YRPW motif
HITS-CLIP: high-throuput sequencing and cross-linking immunoprecipitation
HSC: hematopoietic stem cells
Irf8: interferon regulatory factor-8
JNK: c-Jun N-terminal kinase
MafB: v-Maf musculoaponeurotic fibrosarcoma oncogene family member B
M-CSF: macrophage colony-stimulating factor
Msi: Musashi
NFATc1: nuclear factor of activated T cells 1
NF-*κ*B: nuclear factor-*κ*B
NICD: Notch intracellular domain
PARP: poly (ADP-ribose) polymerase
PI3K: phosphatidylinositol 3-kinase
RANKL: receptor activator of NF-*κ*B ligand
RBPJ*κ*: recombinant recognition sequence binding protein at the J*κ* site
7′AAD: 7-aminoactinomycin-D
shRNA: short hairpin RNA
TRAP: tartrate-resistant acid phosphatase
